# Initial Stage of the COVID-19 Pandemic: A Perspective on Health Risk Communications in the Restaurant Industry

**DOI:** 10.3390/ijerph191911961

**Published:** 2022-09-22

**Authors:** Xi Wang, Liang Tang, Linan Zhang, Jie Zheng

**Affiliations:** 1 Culture, Creativity and Management, School of Culture and Creativity, BNU-HKBU United International College, Zhuhai 519085, China; 2 Department of Apparel, Events and Hospitality Management, College of Human Sciences, Iowa State University, Ames, IA 50011, USA

**Keywords:** Wilson’s theory of information behavior, health risk communication, customer emotion, COVID-19 pandemic, textual review

## Abstract

Restaurant online review websites have made changes to adapt to customers’ shifting needs during the COVID-19 crisis. Based on information behavior theory and social penetration theory, the present study investigated the changes in customers’ emotions and how the volume of online reviews as an indication of sales is impacted by the instructional (i.e., with quantitative variables) and emotional (i.e., with qualitative variables) information on review websites. By comparing the same month (January–April) during 2017–2020, positive sentiment experienced a plunge, while negative sentiment showed an upsurge in April 2020. The volume of reviews was impacted by five quantitative variables (i.e., confirmed COVID-19 case number, food delivery option, takeout option, delivery fee, and delivery time) and seven qualitative variables (i.e., anticipation, fear, trust, anger, disgust, joy, and sadness). This study provides new insight into understanding information content on review websites during the crisis (e.g., pandemic) from the perspective of health risk communication.

## 1. Introduction

In the initial stage (January–April 2020) of the COVID-19 pandemic, the U.S. restaurant industry experienced unparalleled challenges [1]. The Centers for Disease Control and Prevention (CDC) confirmed the first coronavirus case in the U.S. on 21 January 2020. The net sales of restaurants plummeted from $65.4 billion in February to $30 billion in April 2020, a decrease of 54.1% [2]. Social distancing became one of the crucial measures implemented to effectively prevent the spread of COVID-19 [3]. Accordingly, from March 2020, many restaurants (e.g., McDonalds) closed dine-in areas and instead only provided delivery and pickup service. The top food delivery companies (e.g., Postmates, DoorDash) changed their business models and promoted contactless delivery [4].

Although the consumption pattern change at the initial stage of the pandemic can be explained by various reasons (e.g., governmental lockdown policies, news media, CDC’s guidance), the dominant reason from a psychological perspective was customers’ uncertainty and anxiety regarding infection risks at restaurants. For instance, with regard to the dining behavior of customers, due to the concerns arising from the pandemic, a majority of customers subjectively developed unfavorable attitudes toward going outside [5]. However, when customers had no choice but to go to public locations, they would involuntarily have a sense of danger about the infection, which is likelier to result in significant mental stress as well as negative emotions (e.g., confusion, fear, and anxiety) [6]. Moreover, such mental stress and negative emotions can also make a difference in determining their dining behaviors. For instance, rather than going outside, some chose to utilize food delivery or pickup services [7].

The psychological uncertainty and anxiety of customers toward the risk of infection can be explained by Wilson’s model of information behavior [8]. Regarded as the foundational theory of risk communications in the healthcare field, Wilson’s model of information behavior indicates that acquiring information when customers face health risks has both instrumental (i.e., “doing something” about a potential threat) and emotional (i.e., affective consonance) values [9]. The instructional value of health risk communications is dominantly conveyed in numerical information presented by government offices (e.g., reporting of COVID-19 confirmed cases at a geographic location in a specific period of time) and individual businesses (e.g., information on extra services, such as out-of-store options of a restaurant on review websites) at the initial stage of the pandemic. The present study identified six quantitative variables, with one from the CDC (i.e., number of confirmed COVID-19 cases in a specific city on a monthly basis) and five from Yelp (i.e., delivery option, takeout option, delivery fee, delivery time, and cuisine preparation time). The emotional value of health risk communications is mainly conveyed through textual reviews from previous customers. In the setting of review websites, a reader echoes emotions expressed in a review, and consequently makes patronage decisions consistent with those of other reviewers. Such emotional echoes offer effective social support when people experience uncertainty and anxiety about health [10].

From the perspective of emotions, mixed emotions are usually generated when negative sentiments strengthen (e.g., after a negative event or occurrence like the COVID-19 crisis) and are blended with enduring positive sentiment [11]. A recent survey on public emotions toward the COVID-19 pandemic indicated that 69% of respondents reported mixed emotions, but only 3% expressed solely negative sentiments [12]. Therefore, instead of one-dimensional scalability, the present study tested customers’ multi-dimensional emotions extracted from textual reviews based on Plutchik’s emotional wheel [13].

The volume of reviews is an indicator of restaurant sales. The volume of reviews during a period (e.g., specific months during the pandemic) predicts the success of a business [14] and impacts prospective customers’ behaviors for two reasons. First, more reviews indicate the popularity of a business, which has widely been used as an indicator of sales [15]. One study found that “consumers’ behavior is influenced by concerns over what others might think of them or how others might act toward them as a function of their product choice and usage.” [16] Thus, many customers make purchase decisions with the rationale that the business has many other patrons. Second, readers view a large bundle of review content as more instructive. The more instructive the review content is, the more satisfactory readers are [17]. Thus, this study used the volume of reviews to benchmark the effectiveness of customer’s health risk communications, where the more reviews received by a certain restaurant, the higher the sales performance of the restaurant was. We tested how the volume of reviews is influenced by instructional and emotional information on review websites at the initial stage of the COVID-19 pandemic.

In addition to the many studies investigating people’s emotional reaction to public health issues, social media has widely disseminated the negative emotions of people facing the haze of a public health issue [18]. One study investigated the effects of emotion regulation on individual’s mental health condition [19]. However, the literature neither examines in depth the fluctuation in customer emotion along with a monthly timeline, nor explores the effects of customer emotional responses applying multi-dimensions (trust, disgust, joy, sadness, surprise, anticipation, anger, fear) when confronting a critical public health issue in the U.S. context. Therefore, the purposes of the present study were as follows: (1) assess the trend of emotional changes conveyed via textual reviews posted by restaurant customers by comparing data between January and April 2020 and the same months within the time period 2017–2019; (2) investigate how the volume of reviews is impacted by the instructional and emotional variables on review websites in January–April 2020. This research enriched the body of theories of health risk communications by investigating a new setting (i.e., review websites during the pandemic). Wilson’s information behavior is first applied to understand the role and strategies of review websites in communication with the public when the relevant information is extremely deficient in the initial stage of the pandemic. It also bridges the gap between communications theories and big data analytics of instructional and emotional information on review websites. The results provide review websites and restaurant owners with effective persuasive strategies during a pandemic. In particular, the refined emotional communications of textual reviews are expected to reduce customers’ uncertainty of patronized restaurants, which not only maximizes future outcomes (i.e., promotes healthy behaviors during the pandemic) but also defeats affective stress.

## 2. Literature Review

### 2.1. Customer Review Websites as Health Risk Communication Channels

Risk communication is a crucial area for government offices, organizations, and businesses when dealing with pandemics [20]. Specifically, customers always try to seek information to reduce health risk in highly uncertain situations no matter whether they want to pick up from a restaurant or order food delivery online during the COVID-19 period. Information seeking was defined as active efforts through which an individual searches for information to meet informational needs or goals, which are initiated in response to situational uncertainty [21]. Situational uncertainty occurs “when a particular event cannot be adequately structured or categorized because it is marked by unpredictability, ambiguity, and a lack of information” [22].

**Figure 1:**
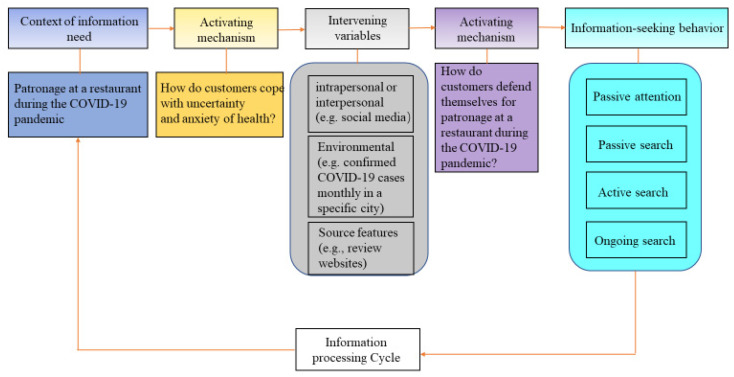
Revised Wilson’s model of information behavior.

Wilson established a theoretical framework of information behavior (Figure 1) [8], which has been widely used to understand risk communications in the healthcare field. In the first stage, an individual is set in the context of information need (e.g., humans had highly limited knowledge of COVID-19 at the initial stage of the pandemic). The individual is eager to cope with stress and anxiety in the second stage. In the third stage, intervening variables (e.g., environmental, source characteristics) play a role in the individual’s responses. The individual activates a self-defense mechanism in the fourth stage. Consequently, the individual’s information-seeking behavior is initiated (e.g., search information on review sites). In both the third and fourth stages, pursuing interpersonal and social support motivates the individual to seek information, which thus determines that a social network such as Yelp is a crucial channel for health risk communications during the pandemic.

Two main approaches are used to communicate health risk information [10]. The first approach is a numerical probability-based approach, which utilizes numerical information for communicating the ratio of a given risk materializing (e.g., a statement like “an individual has a one in 400 chance of developing stomach cancer in three years”) [23]. At the initial stage of the pandemic, there was no information available about the numerical probability for a customer to be infected in restaurants. The monthly number of confirmed COVID-19 cases in a specific city was the primary indicator of risk status [24,25], which was used as a quantitative variable in a numerical probability-based approach.

The second approach to risk communications is to address the informational context to help people comprehend threats [23]. The feature of a contextualized approach for a specific illness is to offer information about its antecedents and/or consequences [26]. Different intervention methods to cure the illness are also included in the contextualized approach [10]. In the setting of restaurants, the alternative strategies (e.g., “out-of-store service” options in this study) serve as intervention methods to respond to the pandemic. Furthermore, textual reviews written by previous customers emphasize the exclusive context (i.e., an individual restaurant). Both instructional (i.e., quantitative variables) and emotional (i.e., qualitative variables) values conveyed in numerical probability and contextualized approaches are crucial in health risk communications [27].

### 2.2. Quantitative Attributes on Customer Review Websites and Their Influences on Volume of Reviews

Quantitative variables refer to any quantity-related aspects of content available in a restaurant’s review on Yelp (e.g., numerical rating) [28]. Quantitative variables potentially relevant to the pandemic were used in this study, including delivery option, takeout option, delivery fee, cuisine preparation time, delivery time, and confirmed COVID-19 cases.

Due to shelter-in-place and social distance mandates implemented in many U.S. locations during the pandemic, many customers had to choose the delivery and takeout options restaurants offer, and it was the “out-of-store service” that was crucial to a customer’s entire dining experience [29]. On the other hand, restaurants also started providing delivery services in response to the necessity of meeting safety concerns, such as maintaining social distance [30]. Therefore, the delivery service (H1) is a solution to satisfy both parties. Meanwhile, according to one study, restaurants with delivery services made a difference when customers evaluated their dining options, selected restaurants, and ordered foods/drinks following the outbreak of COVID-19 [31]. Restaurants can either partner with third-party delivery businesses (e.g., DoorDash) or offer an in-house delivery fleet. Regardless of who is responsible for the delivery, the food delivery option of restaurants is available for customers, which has positively reduced customer anxiety and uncertainty about infection risks [32]. Furthermore, an effective delivery service perceived positively by customers not only reinforces the dining experience but also increases satisfaction, and either one of these reasons positively influences the volume of online reviews. 

The takeout option (H2) refers to an alternative choice provided by restaurants whereby customers carry their ordered foods to a comfortable place outside of, or at, home. It enables customers to avoid the hygiene hazards associated with public interactions with strangers [33]. Due to the pandemic, the takeout option has become the most preferable choice for many customers [34]. In the same manner as delivery, the takeout option has an impact on customer satisfaction and affects customers’ intention to post online restaurant reviews [32].

Regarded as an additional charge when customers choose online food delivery services, the delivery fee (H3) is also believed to have a significant influence on the entire dining experience [35]. In general, restaurants charge a food delivery fee based on the distance of the delivery, and customers in most cases also accept this distance-based charge structure. However, if the delivery fee charged by a certain restaurant is higher than other restaurants or third-party services, it severely affects customers’ perceived fairness related to the delivery fee [36], which counteracts the revisitation intention to that restaurant and negatively impacts the volume of reviews [37].

Cuisine preparation time (H4) is determined by the kitchen’s capacity. As discussed in previous studies, customers who order food delivery rather than dining outside or cooking for themselves accept a high premium on the speed of the dinner [38]. Although due to the differences in the restaurant type (e.g., fast food vs. fine dining), a variance exists in food preparation time. When a customer selects a certain type of restaurant, he/she has already been provided with a preliminary expectation of the food preparation time [39]. For instance, customers choosing fast food normally expect their orders to be prepared and served more quickly than those who would like to have a fine dining experience [39]; however, generally speaking, the shorter the cuisine preparation time of restaurants, the higher the pleasure level retained by customers [40]. Considering the significance of customers’ pleasure as another expression of satisfaction, it is assumed that the longer cuisine preparation time negatively affects the volume of reviews. The delivery time (H5) is dependent on the distance from the restaurant to the drop-off site, efficiency of the delivery staff, and food preparation time [41]. As discussed above, the food delivery speed is a critical concern for customers who choose a delivery service [41]. Moreover, if it takes too long before being delivered, the taste, flavor, and presentation of food may worsen [42], which may further destroy the dining experience and influence the satisfaction level of customers [43]. Therefore, based on these findings, a longer delivery time is believed to have a negative effect on the volume of reviews.

Another quantitative variable tested in the present study was the number of confirmed COVID-19 cases (H6) in a specific city on a monthly basis. A confirmed case is termed an individual with lab confirmation of COVID-19 [24]. The number of confirmed COVID-19 cases represents the severity and spread of the pandemic. These numbers are metrics adopted by national, state, and local authorities to decide which ventures and activities can be relaunched and what restrictions should be enforced [44]. Accordingly, the closure and restrictions of service options at a restaurant are highly impacted by confirmed COVID-19 cases in a specific city [45]. Meanwhile, the increasing number of confirmed COVID-19 cases decreases people’s willingness to visit public areas (e.g., restaurants) [5], and the reduced restaurant visitors further have a negative effect on the volume of reviews generated.

Based on the discussions above, the following hypotheses were proposed:

**Hypothesis** **1** **(H1).** *The delivery option has a positive impact on the volume of reviews*.

**Hypothesis** **2** **(H2).** *The takeout option has a positive impact on the volume of reviews*.

**Hypothesis** **3** **(H3).** *The delivery fee has a negative impact on the volume of reviews*.

**Hypothesis** **4** **(H4).** *Cuisine preparation time has a negative impact on the volume of reviews*.

**Hypothesis** **5** **(H5).** *Delivery time has a negative impact on the volume of reviews*.

**Hypothesis** **6** **(H6).** *Confirmed COVID-19 cases has a negative impact on the volume of reviews*.

### 2.3. Qualitative Attributes on Customer Review Websites and Their Influences on Volume of Reviews

The qualitative variables refer to textual content, specifically textual reviews on Yelp [46]. Compared to the aforementioned quantitative variables, textual reviews are a crucial channel of conveying emotions. Emotion is a subjective state of mind, which involves reactions to events happening in the external environment (e.g., pandemic) [47]. Emotional communications offer social support when people experience health anxiety [10]. In addition to the general positive vs. negative sentiment analysis, we use several multidimensional frameworks of emotions applied in the psychology discipline in other areas.

**Figure 2:**
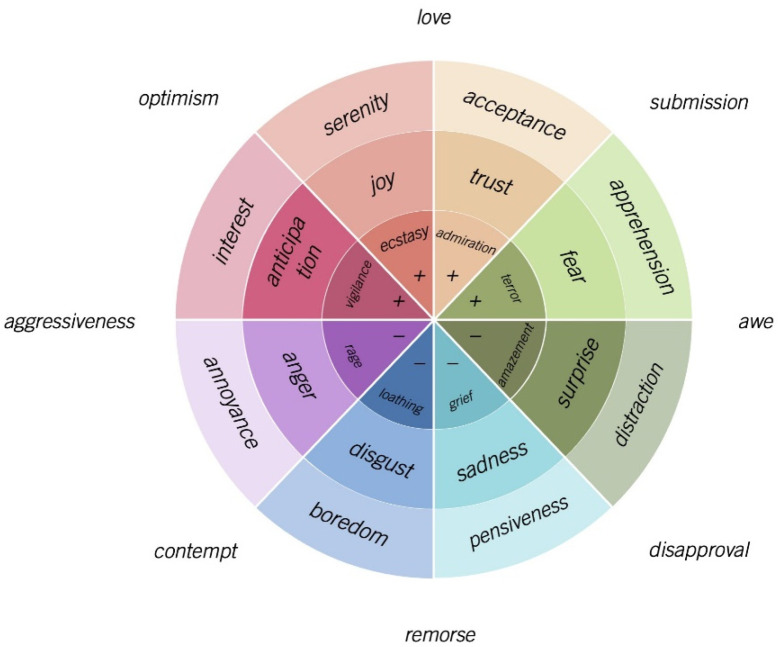
Revised Plutchik’s Emotion Wheel.

Referring to Figure 2, Plutchik’s Wheel of Emotions indicates that the extent of emotional intensity amplifies from the outside of the emotion wheel to the center of the emotion wheel [13]. For instance, fear goes from apprehension (weakest) to terror (strongest), and trust goes from acceptance (weakest) to admiration (strongest). An emotion dyad exists by mixing any two adjacent emotional dimensions in the wheel (e.g., disgust and anger are combined to generate contempt). Tertiary sensations may also exist by mixing three or more emotional dimensions, which are not illustrated in Figure 2.

Plutchik’s Wheel of Emotions was adopted in the present study for three reasons. First, Plutchik’s Wheel of Emotions is a well-established theory in the psychological field, which has been widely utilized in analyzing the perspective of consumer psychology such as maintaining the customer trust level in a business [48] and increasing the business attractiveness to the customer [49]. In the hospitality industry, Plutchik’s Wheel of Emotions was applied to investigate the psychological impression of the customer to evaluate the overall impression of their dining experience [50].

Second, Plutchik’s Wheel of Emotions introduced more insights into the emotion analysis. In addition to the six distinct emotion model developed by Ekman [51] or three categorial emotion model proposed by Francisco and Gervas [47], Plutchik’s Wheel of Emotions extended the traditional emotion concept and further considered two more emotions (i.e., trust and anticipation) so as to formulate a framework consisting of eight-emotional dimensions, which not only can discretely evaluate every individual emotion, but also can compare emotional effects from four opposite pairs [13].

Third, this framework has been extensively applied to analyze emotions in textual content. People present their emotions and deliver messages via multiple methods; significantly, with the rise of social media, social media has become a trending platform to deliver emotions, for instance, via online customer reviews [52], micro-blogs [53], and social media messages [54]. Moreover, people adjust their social lives using social media platforms since in-person communication was discouraged during COVID-19 [55]. Text is one of the prevailing methods to deliver emotions. Therefore, the Plutchik’s Wheel of Emotions serves as a sound theoretical foundation for this study. Two polars of sentiments (positive vs. negative) and eight emotional dimensions (trust, disgust, joy, sadness, surprise, anticipation, anger, fear) expressed in customer text reviews on Yelp were integrated and investigated, and the following hypotheses were proposed.

**Hypothesis** **7a** **(H7a).** *Joyexpressedin reviews has a positive impact on the volume of reviews*.

**Hypothesis** **7b** **(H7b).** *Anger expressed in reviews has a positive impact on the volume of reviews*.

**Hypothesis** **7c** **(H7c).** *Trust expressed in reviews has a positive impact on the volume of reviews*.

**Hypothesis** **7d** **(H7d).** *Anticipation expressed in reviews has a positive impact on the volume of reviews*.

**Hypothesis** **8a** **(H8a).** *Sadness expressed in reviews has a negative impact on the volume of reviews*.

**Hypothesis** **8b** **(H8b).** *Fear expressed in reviews has a negative impact on the volume of reviews*.

**Hypothesis** **8c** **(H8c).** *Disgust expressed in reviews has a negative impact on the volume of reviews*.

**Hypothesis** **8d** **(H8d).** *Surprise expressed in reviews has a negative impact on the volume of reviews*.

## 3. Methodology

### 3.1. Data Collection

Data was collected from Yelp.com between 4–10 May 2020. The information of restaurants in three cities (i.e., New York City, Los Angeles, and Chicago) was selected as the sample because they are the top three cities with the highest population in the country based on the U.S. Census Bureau report [56]. Since we targeted the initial stage of the COVID-19 crisis, textual reviews posted between January and April 2020 were filtered for further analysis. The data were selected from January to April because customers changed consumption modes to respond to COVID-19 in March and April. A number of restaurants shut down, closed the dining section, and changed their mode of operation to delivery and pickup only [7]. The data between the first two months (i.e., January and February) and the next two months (i.e., March and April) were compared to show the difference before and after the outbreak of COVID-19.

In this study, March 2020 was regarded as the critical point for the outbreak of the pandemic for three reasons. First, as officially announced by the CDC, the pandemic report indicated that the number of new cases increased significantly in the month of March [57]. Second, the present study referred to Schuchat et al. [58], who adopted the period of 24 February–21 April 2020 in investigating the initial spread of COVID-19 in the U.S. Therefore, according to the three aforementioned reasons, March 2020 was regarded as the critical point for the start of the pandemic. Moreover, in addition to the initial stage of COVID-19, customer review information posted during the same months (January–April) in previous years from 2017 to 2019 was also extracted in order to conduct comparative analyses. In addition to the textual reviews, the quantitative information of individual restaurants on Yelp was also collected, as discussed in Section 3.2. After preprocessing the initial dataset (i.e., data cleaning and matching), 356 restaurants and their corresponding 69,581 reviews were selected for further analysis.

### 3.2. Variables

The volume of reviews in January–April 2020 was used as the dependent variable. The independent variables comprised both quantitative measures and qualitative surrogates. Five of the six quantitative measures were collected from the review webpage of each restaurant, including the food delivery option, take-out option, delivery fee, cuisine preparation time, and delivery time. The food delivery option and takeout option were both coded with 1 (yes) or 0 (no). Delivery fees ranged from $0 to $5.99. Cuisine preparation time ranged from 0 to 35 minutes. Delivery time was measured on a scale of 1–6, including less than 20, 20–30, 30–40, 40–50, 50–60, and more than 60 mins. Another quantitative measure was the number of confirmed COVID-19 cases in each of the three cities on a monthly basis.

The qualitative surrogates were the eight dimensions of Plutchik’s emotion wheel. The emotional words were recognized and assessed using Linguistic Inquiry and Word Count (LIWC). Developed by Pennebaker [59], LIWC can help identify diverse cognitive, emotional, and structural components in different kinds of textual contexts, such as formal paragraphs, speech, and online reviews. Through a series of analytic processes, LIWC aims to calculate the percentage of each word in a specific category by comparing it with its own developed dictionary, and then indicates a specific value score for a certain measured category such as the emotion category in the present study [60]. However, due to the limitation of the default dictionary of LIWC, only three specific emotions (i.e., anxiety, anger, and sadness) could be detected in the analysis. Therefore, to achieve the multi-dimensional emotion analysis based on Plutchik’s emotion theory, this study embedded the Word-Emotion Association Lexicon dictionary (EmoLex) in the LIWC software to accurately identify the eight emotional dimensions proposed by Plutchik [13]. Developed by the National Research Council (NRC) Canada, EmoLex specializes in processing the eight basic emotions including anger, joy, anticipation, sadness, disgust, surprise, fear, and trust [61]. The implications of EmoLex focusing on sentiment and emotion analyses have been extensively verified in diverse contexts, such as online comments on the hospitality and restaurant industry [62,63], general user-generated social media content [64,65], and even well-being, and pandemic response [66,67].

Four control variables were adopted: city, month, restaurant price, and total volume of reviews for a specific restaurant available on Yelp. Restaurant price was measured from “1” to “4” (i.e., “$” to “$$$$”). The total volume of reviews for a specific restaurant means the exact count of reviews by the date of data collection. City was coded as 1 (New York City), 2 (Los Angeles), and 3 (Chicago). Month was coded as 1 (January), 2 (February), 3 (March), and 4 (April).

### 3.3. Poisson Regression Model

**Figure 3:**
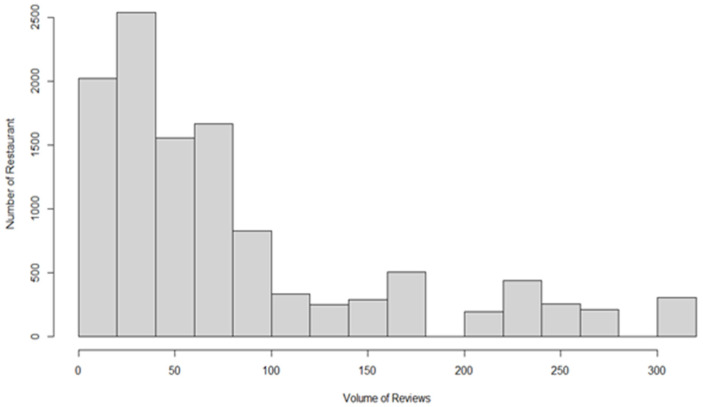
Distribution of volume of reviews.

Poisson regression was used in the data analysis mainly for two reasons. First, the dependent variable satisfied the fundamental assumption of Poisson regression that the respondent variable should be a discrete count variable rather than a continuous attribute [68]. Since the dependent variable volume of reviews in this study was actually collected by counting the number of reviews related to a certain restaurant posted on Yelp, it satisfied the fundamental assumption to apply Poisson regression. Second, with regard to the distribution of the dependent variable in Figure 3, although the distribution of the dependent variable was not normally distributed, it adequately matched the Poisson distribution and satisfied the second assumption for the Poisson regression. Concerning the present analysis, compared with the traditional ordinary least squares method, Poisson regression is more effective and precise in measuring such over-dispersed distribution of a count model [69]. Therefore, Poisson regression was applied in the current study for data analysis.

## 4. Results and Discussions

### 4.1. Changes in Sentiment Polarity and Individual Emotions by Month

**Figure 4:**
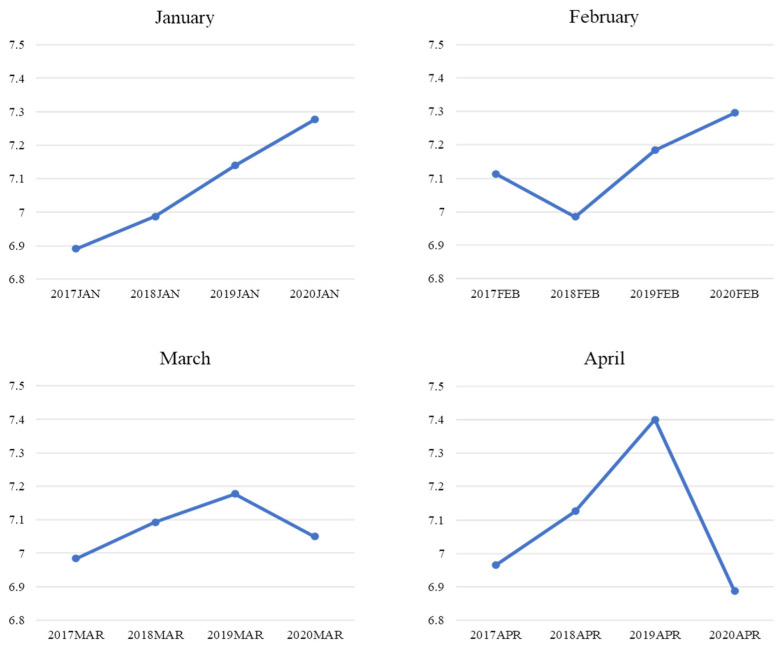
Mean comparison of positive sentiment by month.

**Figure 5:**
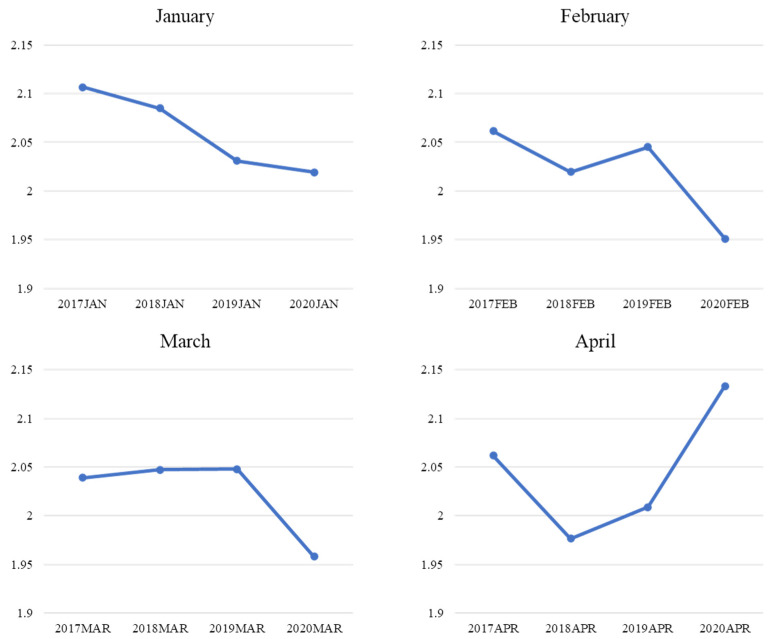
Mean Comparison of Negative Sentiment by Month.

The purpose of this section is to outline the objectives of this study. Figure 4 reveals the changes in positive sentiment extracted from customer reviews in specific months (i.e., January–April) from 2017 to 2020. Generally, positive sentiment showed an uptrend in January and February with the exception of only February 2018. However, a downswing in positive sentiment was observed in March, and a slump of the curve even occurred in April 2020. These findings related to the fluctuation in negative sentiments starting in March 2020, coinciding with the curve of the surge in the confirmed cases of COVID-19 in the U.S. [70]. Meanwhile, in March, the number of new cases per 100,000 residents was 56, but by the end of April, this number had risen to 200 and above [70]. Therefore, it was reasonable to assume that customers would be anxious and feel disturbed under such circumstances; thus, they would be likelier to generate more negative emotions that affected uncertainty. Meanwhile, contrasting results were found in the trend in negative sentiment extracted from customer reviews (Figure 5). Generally, negative sentiment demonstrated a downtrend in January, February, and March with the exception of only February 2019. However, an upsurge of negative sentiments was observed in April from 2017 to 2020.

A series of T-tests (Table 1) were conducted to further verify the difference between the two affection extremes in a specific month prior to (i.e., 2017–2019) and during the pandemic (i.e., 2020). The average of an affective extreme in an identical month from 2017 to 2019 was used to compare it with the corresponding month in 2020. The T-test results were consistent with Figure 4 and Figure 5. A comparison of the two sentiment extremes in April is presented as an example. A significant difference between the positive affection in 2020 and the average of those from 2017 to 2019 was found (t = −2.03; *p* = 0.02). This indicated that customers showed a lower extent of positive affection in reviews in April 2020 compared to the same period in previous years. The significant difference between negative affection in 2020 and the average of that from 2017 to 2019 was identified as consistent (t = 1.83; *p* = 0.03). This means that customers expressed a higher level of negative affection in reviews in April 2020 compared to the same period in previous years.

**Figure 6:**
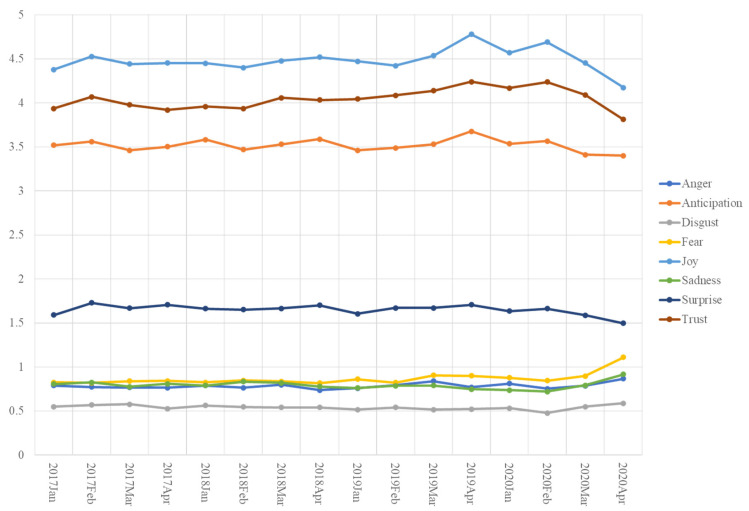
Eight Emotions Comparison by Month.

Eight emotional dimensions noted in January–April from 2017 to 2020 are demonstrated in a timeline in Figure 6. An apparent decrease was observed in the dimensions of joy, trust, anticipation, and surprise from February 2020. In contrast, fear, sadness, anger, and disgust showed a significant increase from February 2020.

A series of T-tests (Table 2) were further applied to verify the difference in individual emotional dimensions in a specific month prior to (i.e., 2017–2019) and during the epidemic (i.e., 2020). The average of an individual emotional dimension in an identical month from 2017 to 2019 was compared to its average in the corresponding month in 2020. The T-test results were consistent with the visual demonstration presented in Figure 6. A comparison of the emotional aspects in April was adopted as an example. The significant difference of four individual emotional dimensions between April 2020 and the average of those in April from 2017 to 2019 was identified [joy (t = −3.52, *p* = 0.00); anticipation (t = −2.07, *p* = 0.02); surprise (t = −3.30, *p* = 0.00); trust (t = −2.46, *p* = 0.01)]. This meant that customers expressed a lower level of joy, anticipation, surprise, and trust in reviews in April 2020 compared to the same period in the previous years. A significant difference in the other four individual emotional dimensions between April 2020 and the average of those in April from 2017 to 2019 was also found [anger (t = 2.75, *p* = 0.00); sadness (t = 3.47, *p* = 0.00); disgust (t = 1.70, *p* = 0.04); fear (t = 5.66, *p* = 0.00)]. This indicated that customers showed a higher extent of anger, sadness, disgust, and fear in reviews in April 2020 compared to the same period in previous years. 

### 4.2. Regression Analysis

In order to realize the second research objective of investigating how the volume of reviews is impacted by the instructional and emotional variables on review websites during the initial stage of COVID-19, only information from the beginning of 2020 (January–April) was filtered out from the dataset for further analysis. As discussed in Section 3.3, a Poisson regression was performed in this study because the dataset followed a Poisson distribution and the characteristic of the dependent variable was a counted event. The regression model was acceptable with a log-likelihood value = −84412.09, df = 19, and AIC = 168862. The results of all the variables of interest are presented in Table 3. Except for food preparation time (H4 rejected), all other quantitative measures had significant influences on the volume of reviews. Specifically, confirmed COVID-19 case number (β = 0.0001, *p* = 0.0000; H6 rejected) and food delivery option (β = 0.0131, *p* = 0.0256; H1 accepted) had positive effects, while takeout option (β = −0.1136, *p* = 0.0000; H2 rejected), delivery fee (β = −0.2475, *p* = 0.0000; H3 accepted), and delivery time (β = −0.0436, *p* = 0.0000; H5 accepted) had negative effects on the review volumes.

Among the eight qualitative surrogates (i.e., eight emotional dimensions), anticipation (β = 0.0063, *p* = 0.0000; H7d accepted), fear (β = 0.0623, *p* = 0.0000; H8b rejected), and trust (β = 0.0118, *p* = 0.0000; H7c accepted) had positive effects, while another four, anger (β = −0.0055, *p* = 0.0002; H7b rejected), disgust (β = −0.0198, *p* = 0.0000; H8c accepted), joy (β = −0.0081, *p* = 0.0000; H7a rejected), and sadness (β = −0.0294, *p* = 0.0000; H8a accepted) had negative impacts on the volume of reviews. Only the dimension of surprise (H8d rejected) showed an insignificant effect on the volume of reviews. Meanwhile, all control variables were identified as significant in the regression analysis.

The number of confirmed COVID-19 cases on a monthly basis in a specific city had a positive influence on the volume of reviews. One reason is delays in customers’ responses toward COVID-19 at the initial stage of the pandemic. The other reason is that the cities used in this study were among the largest cities in the U.S. Community-based confirmed COVID-19 cases may be more relevant to local restaurants, which can be a direction for future studies.

Cuisine preparation time did not have a significant impact on the volume of reviews because customers had reasonable expectations of cuisine preparation time during the pandemic. The takeout option had a negative impact on the volume of reviews. A survey conducted by WebMD in early May 2020 indicated that 26% of the respondents experienced trauma from COVID-19, 25% were scared of entering stores, and 15% were reluctant to leave their shelters [71]. Thus, it seems that at the initial stage of the pandemic, customers were anxious, felt uncomfortable, and were not prepared to depart from home and pick up packaged food from a restaurant.

Surprise was not an influential factor in the volume of reviews at a restaurant. Surprise conveys a reviewer’s psychological status engendered by unpredicted circumstances. From a broader perspective, surprise is often linked to other affections, such as neutral, pleasurable, and disagreeable [72]. Thus, surprise may not convey an exact feeling of a reviewer, and did not predict restaurant popularity (i.e., volume of reviews). Joy negatively influenced the volume of reviews. When the general public faced uncertainty and health risks during the pandemic, they were more inclined to view the reviews addressing happiness and enjoyment as fabricated. Fear and anger are a pair of opposite dimensions (i.e., anger as a positive emotion vs. fear as a negative emotion) in Plutchik’s emotional wheel [13]. Although anger is included in the extreme of positiveness, it conveys an undesirable reaction. In contrast, for the general public in the face of uncertainty, fear becomes a shared feeling and is not unwanted. 

## 5. Conclusions

By comparing online restaurant reviews in the same months from three different years, textual reviews in 2020 showed an uptrend from the perspective of positive sentiment in January, but a downswing in March and even a plunge in April. In contrast, negative sentiments embedded in textual reviews showed a downtrend from January to March but an upsurge in April 2020. More specifically, compared to the average number in the same month during 2017–2019, customers demonstrated a lower extent of joy, anticipation, surprise, and trust and a higher extent of anger, sadness, disgust, and fear in textual reviews in April 2020. Furthermore, the volume of reviews was significantly influenced by five quantitative variables (i.e., confirmed COVID-19 case number, food delivery option, takeout option, delivery fee, and delivery time) and seven qualitative variables (i.e., anticipation, fear, trust, anger, disgust, joy, and sadness).

Meanwhile, the restaurant price level represented by the number of "$" was also considered in this study to explore the effect on the volume of reviews received. Of the total sample, 356 restaurants involved in this study, the largest number of restaurants were tagged with the "$$" tag (253, 71.1%), while 57 (16.0%), 38 (10.7%) and 8 (2.2%) restaurants were tagged with "$", "$$$", and "$$$$", respectively. On the Yelp platform, the four different price labels represent the restaurant's per capita spending of "under $10", "$11–30", "$31–60", and "over $61”. Through labeling price with “$”, customers are able to visually understand the restaurant's price range and make a quicker decision. According to the analysis, a positive relationship was identified between a higher price level and the number of reviews. From the perspective of the restaurant, the higher the spend, the better the restaurant’s ability to control the quality of food and service, which often provides an impressive experience for the customer, thus attracting them to leave a review on Yelp after the meal. While from a customer perspective, celebrating special events or anniversaries is often one of the motivations for visiting a restaurant with high charges, which also drives customers to share their dining experience and can explain why restaurants with higher spending levels tend to attract more online reviews.

In general business, an effective communication tool would help both businesses and consumers to share and communicate information. As for the restaurant industry, an effective communication tool (e.g., Yelp) helps business adjust strategies and improve their profit. On the other hand, the effective tool also allows consumers to share reviews and express emotions. From February 2020, people experienced emotional shifting during the COVID-19 period, and negative sentiments like fear, sadness, anger, and disgust. Based on the study results, consumers’ emotions of anticipation, trust, and fear had a positive effect on the volume of reviews by analyzing textual reviews from the communication tool (online website).

What is more, food delivery service had a positive influence on the volume of reviews, which compared with other options that restaurants provided during COVID-19. The food delivery option experienced rapid development during the tough conditions. Many restaurants shifted parts of their businesses to provide delivery service. Online review websites like Yelp serve as an efficient communication platform that helps restaurants track consumer preferences (e.g., using quantitative variables—food delivery option, takeout option etc.) and analyze corresponding emotion expressions (e.g., using qualitative variables—fear, joy, etc.) so as to help restaurant businesses formulate new strategies to respond to COVID-19.

This research provides important theoretical contributions from three perspectives. First, the COVID-19 pandemic is an unprecedented and ongoing phenomenon. To date, no recent studies have applied the theories of health risk communications to understand the role of customer review websites in response to the pandemic in the general business administration discipline, especially in the hospitality and tourism fields. Customer review websites are a dominant platform for many restaurants, especially mom-and-pop ones, to communicate updated information relevant to the pandemic to prospective customers. The present study identified qualitative and quantitative variables in response to COVID-19 on review websites based on Wilson’s model of information behavior, twofold values along with the health information seeking behavior (i.e., instrumental values and emotional values) [9], and dual information communication approaches (i.e., numerical probability-based and contextualized approach) [10]. These psychological foundations are urgent and critical for the new domain of hospitality and tourism research relevant to the pandemic.

Second, this study pioneered the use of social penetration theory to explain the role of emotions in interpersonal decision making in social networks in the hospitality and tourism field. It is a crucial theoretical foundation to understand the emotional support a reader can gain from textual reviews during the pandemic, which further predicts their attitude and behavior. Furthermore, instead of positiveness–negativeness extremes, the authors realized that people experienced mixed emotions during the pandemic. Mixed emotions offer the theoretical foundation to understand customers’ conflicted thoughts and perceptions about a dilemma. For example, customers might not have been accustomed to social distancing but obeyed it for public health. Or customers may enjoy the novelty and flexibility of curbside pickup for the delivery, even though it is disruptive to the dining-out experience.

Third, the present study contributed to the understanding of customers’ interpersonal decision making, which is different from the intrapersonal perspective in most previous studies in the field of hospitality and tourism. In this field—a people industry—customers show the interpersonal feature of emotion with which an information sender’s (e.g., a previous customer) emotions transfer to the receiver (i.e., a prospective customer). In the setting of review websites, emotions embedded in communications can assist individuals in navigating social interactions by offering information about others’ motivations and dispositions, and lead to the generation and maintenance of healthy and productive social relationships. Keltner and Haidt summarized three functions of emotions in interpersonal decision making: assisting an individual’s comprehension of the other party’s emotions, beliefs, and intentions; imposing a cost on the other party’s actions; and arousing complementary, reciprocal, or shared emotions in the other party [73]. By mixing all three functions, the emotions expressed by reviewers transfer to the moods or feelings of readers, which further influence their patronages.

This study also provides plenty of implications for practitioners in both government offices and food service businesses. First, the present study offers an alternative tool for government offices to conduct a statewide or even countrywide examination of customers’ perceptions toward restaurant offerings and services during the pandemic. With the COVID-19 social-distancing measures, customers’ dining-out behaviors had to change overnight. Accordingly, the profitability of restaurants has taken big hits. Industry practitioners have had limited time to develop strategies to design a new normal for restaurants, as jobs and revenues of the industry were cut significantly or impacted severely. Compared to large-scale surveys and interviews, big data analytics of customer reviews can be immediately available and provide rich information for government offices and industry organizations seeking to understand the general public’s perceptions and experience of adaptive practices by a restaurant, and accordingly offer guidance for healthy eating and formulate policies and regulations for the restaurant industry. Analysis of customer reviews effectively decreases costs and generates real-time results about the general public’s dining-out experience during the pandemic.

Review websites served as health-risk communication channels during the pandemic. Thus, the designers of review websites are advised to adjust content to satisfy the informational and affective needs or goals of customers in response to the pandemic. Specifically, customers seek information with both instrumental and emotional value. When we collected data at the initial stage of the pandemic, only “out-of-store service” information on Yelp was relevant to help customers cope with the uncertainty of COVID-19. Protective measures (e.g., mask requirement) continued to be added to Yelp until July 2021. Comprehensive instrumental information should be offered earlier than later. Hospitality and tourism organizations, review websites, and companies should cooperate to establish a system to help businesses, especially mom-and-pop restaurants, cafes, and diners, to establish effective communicative channels with customers about the pandemic in a timely manner. The health risk communication theories discussed in this study addressed the importance of emotional support and social networks. However, only emotional communications are conveyed through context reviews. Review websites are advised to allow individual business owners to post messages to show sympathy, support, and commitment, which can help reduce customers’ uncertainty of risks and encourage their patronage. Meanwhile, direct communicative messages between business owners and customers can enable them to better understand each other’s challenges and difficulties in dining during the pandemic.

The emotional dimensions have distinct impacts on the volume of reviews. It proves the presence of customers’ mixed emotions during the pandemic. Therefore, in the promotions and marketing materials, restaurant owners are advised to echo customers’ mixed emotions rather than convey solely joyful or depressed feelings. Notably, purely negative emotions may disengage people from the goal of overcoming COVID-19 challenges; mixed emotions are likelier to prepare people to respond to uncertain circumstances in flexible ways. In the restaurant industry context, this means that blended emotions motivate customers to actively seek alternative approaches to dining out rather than completely refusing restaurant services during the pandemic. Customers’ mixed emotions are a start for the restaurant industry to explore innovative platforms (e.g., delivery app, check-in app, robot delivery) to envision the new normal.

Textual reviews are viewed as one-way communication. However, effective emotional communications should be two-way or even multi-way, and this is especially important when prospective customers seek social support in the face of considerable uncertainty and risks during a pandemic. Therefore, review websites are advised to allow prospective customers to leave messages to review writers and encourage review writers to respond to these messages with credits rewarded (e.g., free coffee). Moreover, review websites can give their “elite” programs (e.g., elite squad on Yelp, which is a community of passionate writers and photographers) the guidance to address emotional expressions in their reviews.

## 6. Limitations and Future Research

This research has a few limitations. First, Wilson’s framework is a multi-stage model that can be leveraged to present the cognitive, affective, and behavioral process of information seeking in the field of health risk management. This study only tested the stage of information-seeking behavior in the setting of social networks. Future studies are advised to include the antecedents and consequences of information-seeking behavior into the model, which can predictably provide more implications. Second, at the initial stage of the pandemic when our data was collected, review websites such as Yelp did not supplement quantitative variables related to protective measures (e.g., masks required). Thus, the quantitative variables in response to the pandemic used in this study were limited. Future studies that target the middle or late stages of the pandemic are advised to expand the scope of qualitative variables. Third, the study analyzed emotions from context reviews only. Future studies are advised to extract both topics related to the pandemic and their corresponding emotions, which can provide more practical implications for industry practitioners.

Fourth, this study was the first to analyze multiple emotions of customers toward restaurants during the pandemic. However, due to the limitation of LIWC, the dyad or tertiary emotion between several basic emotional dimensions in Plutchik’s emotional wheel was not analyzed herein. Future studies are advised to employ interviews or neuromarketing tools to meet this goal. Fifth, this study identified that fear and anger as a pair of emotions imposed adverse impacts on the volume of reviews compared to the hypotheses. We explained the result in relation to the uncertainty that humans faced at the initial stages of the pandemic. Future research in the hospitality and tourism field is advised to explore further how the role of an individual emotion is changed in a special social context or event (e.g., a pandemic). Sixth, in the regression analysis of the present study, we obtained certain extra findings beyond the objectives related to the COVID-19 pandemic; specifically, the individual emotional dimensions of textual reviews varied month by month before 2020. This might have been caused by seasons, climate, and other external factors. Future studies can investigate this phenomenon further. Seventh, although this study only examined eight emotional aspects, the fundamental mechanism is applicable to comprehend the influence of an even broader span of sentimental components (e.g., 11 emotional aspects suggested by Arnold) [74]. Finally, this study provided a snapshot of customer emotions as well as behaviors from a consumer behavior perspective in the restaurant industry in the early days of the COVID-19 outbreak. Left for future investigation is how different stakeholders (especially restaurants) responded to the situation. We believe that relevant studies on how stakeholders responded to the COVID-19 situation can provide a very favorable reference for future critical health event response measures.

## Figures and Tables

**Table 1:** t-test of Two Affection Extremes between 2020 and the Average of 2017–2019 by Month.

	t-Stat	df	Sig. 1-Tailed	Sig. 2-Tailed	Mean Difference
January					
Positive	4.06	19408	0.00 **	0.00 **	0.26
Negative	−1.69	19408	0.05 *	0.09 *	−0.05
February					
Positive	2.88	17587	0.00 **	0.00 **	0.20
Negative	−2.80	17587	0.00 **	0.01 **	−0.09
March					
Positive	−0.51	17298	0.30	0.61	−0.04
Negative	−2.17	17298	0.01 **	0.03 **	−0.09
April					
Positive	−2.03	15280	0.02 **	0.04 **	−0.28
Negative	1.83	15280	0.03 **	0.07 *	0.12

Note: * *p* < 0.1, ** *p* < 0.05.

**Table 2:** t-test of eight emotions between 2020 and the average of 2017–2019 by month.

	T-Stat	df	Sig. 1-Tailed	Sig. 2-Tailed	Mean Difference
January					
Anger	1.65	19408	0.05 *	0.10	0.03
Joy	2.46	19408	0.01 **	0.01 **	0.13
Anticipation	0.40	19408	0.35	0.69	0.02
Sadness	−2.48	19408	0.01 **	0.01 **	−0.05
Disgust	−0.60	19408	0.28	0.55	−0.01
Surprise	0.50	19408	0.31	0.62	0.01
Fear	1.86	19408	0.03**	0.06 *	0.04
Trust	3.86	19408	0.00**	0.00 **	0.19
February					
Anger	−1.11	17587	0.13	0.27	−0.02
Joy	4.23	17587	0.00 **	0.00 **	0.24
Anticipation	1.40	17587	0.08 *	0.16	0.06
Sadness	−4.67	17587	0.00 **	0.00 **	−0.09
Disgust	−4.42	17587	0.00 **	0.00**	−0.07
Surprise	−0.56	17587	0.29	0.58	−0.02
Fear	0.69	17587	0.24	0.49	0.01
Trust	3.97	17587	0.00 **	0.00 **	0.21
March					
Anger	−0.72	17298	0.24	0.47	−0.02
Joy	−0.50	17298	0.31	0.62	−0.03
Anticipation	−1.80	17298	0.04 **	0.07 *	−0.10
Sadness	−0.20	17298	0.42	0.84	−0.01
Disgust	0.31	17298	0.38	0.76	0.01
Surprise	−2.10	17298	0.02 **	0.04 **	−0.08
Fear	1.21	17298	0.11	0.23	0.03
Trust	0.42	17298	0.34	0.68	0.03
April					
Anger	2.75	15280	0.00 **	0.01 **	0.11
Joy	−3.52	15280	0.00 **	0.00 **	−0.41
Anticipation	−2.07	15280	0.02 **	0.04 **	−0.19
Sadness	3.47	15280	0.00 **	0.00 **	0.14
Disgust	1.70	15280	0.04 **	0.09 *	0.06
Surprise	−3.30	15280	0.00 **	0.00 **	−0.21
Fear	5.66	15280	0.00 **	0.00 **	0.26
Trust	−2.46	15280	0.01 **	0.01 **	−0.26

Note: * *p* < 0.1, ** *p* < 0.05.

**Table 3:** Poisson regression results.

Coefficients:	Estimate	Std. Error	z Value	Pr(>|z|)
(Intercept)	5.0210	0.0113	444.1240	0.0000 ***
Control variables				
Restaurant price	0.0538	0.0033	16.4740	0.0000 ***
Total volume of reviews for a specific restaurant	0.0001	0.0000	148.4730	0.0000 ***
City	−0.1157	0.0018	−63.6490	0.0000 ***
Month	0.0101	0.0018	5.6300	0.0000 ***
Independent variables				
Number of COVID-19 confirmed cases	0.0001	0.0000	−19.3160	0.0000 ***
Delivery option	0.0131	0.0059	2.2320	0.0256 **
Takeout option	−0.1136	0.0070	−16.2110	0.0000 ***
Delivery fee	−0.2475	0.0021	−118.3860	0.0000 ***
Cuisine preparation time	0.0002	0.0003	0.5350	0.5923
Delivery time	−0.0436	0.0017	−25.7160	0.0000 ***
Anger	−0.0055	0.0015	−3.6690	0.0002 ***
Anticipation	0.0063	0.0007	9.3600	0.0000 ***
Disgust	−0.0198	0.0018	−11.2490	0.0000 ***
Fear	0.0623	0.0011	55.1250	0.0000 ***
Joy	−0.0081	0.0007	−10.9160	0.0000 ***
Sadness	−0.0294	0.0016	−18.9350	0.0000 ***
Surprise	−0.0013	0.0010	−1.2700	0.2042
Trust	0.0118	0.0008	14.6900	0.0000 ***

Note: ** *p* < 0.05, *** *p* < 0.01.

## Data Availability

Data used in this study can be obtained by contacting the corresponding author.
